# The Association of ATG16L1 Variations with Clinical Phenotypes of Adult-Onset Still’s Disease

**DOI:** 10.3390/genes12060904

**Published:** 2021-06-11

**Authors:** Wei-Ting Hung, Shuen-Iu Hung, Yi-Ming Chen, Chia-Wei Hsieh, Hsin-Hua Chen, Kuo-Tung Tang, Der-Yuan Chen, Tsuo-Hung Lan

**Affiliations:** 1 Institute of Clinical Medicine, National Yang-Ming Chiao Tung University, Taipei 11221, Taiwan; wthung@vghtc.gov.tw; 2 Department of Medical Education, Taichung Veterans General Hospital, Taichung 40705, Taiwan; 3 Cancer Vaccine and Immune Cell Therapy Core Laboratory, Chang Gung Immunology Consortium, Chang Gung Memorial Hospital, Linkou, Taoyuan 33305, Taiwan; hungshueniu@gmail.com; 4 Department of Medical Research, Taichung Veterans General Hospital, Taichung 40705, Taiwan; ymchen1@vghtc.gov.tw; 5 School of Medicine, College of Medicine, National Yang Ming Chiao Tung University, Taipei 11221, Taiwan; dirac1982@vghtc.gov.tw; 6 Rong Hsing Research Center for Translational Medicine & Ph.D. Program in Translational Medicine, National Chung Hsing University, Taichung 40227, Taiwan; chiaweih@gmail.com (C.-W.H.); shc5555@hotmail.com (H.-H.C.); 7 Division of Allergy, Immunology, and Rheumatology, Taichung Veterans General Hospital, Taichung 40705, Taiwan; 8 Department of Industrial Engineering and Enterprise Information, Tunghai University, Taichung 40705, Taiwan; 9 Translational Medicine Laboratory, Rheumatology and Immunology Center, China Medical University Hospital, Taichung 40447, Taiwan; 10 Rheumatology and Immunology Center, China Medical University Hospital, Taichung 40447, Taiwan; 11 School of Medicine, China Medical University, Taichung 40447, Taiwan; 12 Tsao-Tun Psychiatric Center, Ministry of Health and Welfare, Nantou 54249, Taiwan; 13 Center for Neuropsychiatric Research, National Health Research Institutes, Miaoli 35053, Taiwan

**Keywords:** ATG16L1, haplotype, single-nucleotide polymorphism, autophagy, adult-onset Still’s disease

## Abstract

Adult-onset Still’s disease (AOSD) is a rare autoinflammatory disease, which has elevated autophagosome levels regulated by autophagy-related gene (ATG) expression. We investigated the associations of ATG polymorphisms with AOSD susceptibility, clinical manifestations, and disease course. The six-candidate single-nucleotide polymorphisms (SNPs) involved in autophagy were genotyped using direct sequencing on samples from 129 AOSD patients and 129 healthy participants. The differentially expressed gene products were quantified using PCR and ELISA. Significant linkage disequilibrium was noted in three SNPs of autophagy-related 16-like 1 (*ATG16L1*) gene (rs10210302, rs2241880, and rs1045100). Although the AA/CC/TT haplotype of *ATG16L1* was not associated with the susceptibility of our AOSD patients compared with other haplotypes, those carrying this haplotype had lower mRNA expression levels of LC3-II, reflecting by autophagosome formation (*p* = 0.026). Patients carrying AA/CC/TT haplotype also have a significantly higher proportion of skin rash and a lower proportion of arthritis compared with other haplotypes. The AA/CC/TT haplotype was significantly associated with systemic pattern (odds ratio, 3.25; 95% confidence interval, 1.15–9.14; *p* = 0.026). In summary, the AA/CC/TT haplotype encoded lower levels of autophagosome formation and was associated with a higher proportion of skin rash and systemic pattern of AOSD compared with other haplotypes.

## 1. Introduction

Adult-onset Still’s disease (AOSD) is characterized by spiking fever, skin rash, arthritis, lymphadenopathy, hepatosplenomegaly, variable multisystemic involvement, and increased acute phase reactants [1,2]. It has recently been considered to be an autoinflammatory disease due to its characteristic phenotypes without detectable autoantibodies [3,4]. Although its pathogenesis remains elusive, AOSD is characterized by increased levels of proinflammatory cytokines, such as interleukin (IL)-1β and IL-18 [5,6,7]. We recently revealed that elevated levels of autophagosome formation and autophagy-related gene (ATG) expression both positively correlated with disease activity, suggesting the involvement of autophagy in AOSD pathogenesis [8].

Autophagy is a genetically programmed process that requires ATG proteins. Among them, Beclin-1 is essential for autophagy initiation [9,10]. Moreover, the genetic variants within or near ATG5, which are required for autophagosome formation, may participate in the pathogenesis of systemic lupus erythematosus at multiple levels [11,12]. ATG7 mediates the elongation of the isolation membrane, which culminates in the conversion of microtubule-associated protein one light chain three (LC3-I) to its phosphatidylethanolamine-conjugated form (LC3-II), a process indicating active autophagy [13,14]. Autophagy-related 16-like 1 (ATG16L1) is required to extend phagophores through the ATG5–ATG12–ATG16L1 complex during autophagosome formation [15]. The polymorphisms in human *ATG16L1* have been associated with an increased risk of Crohn’s disease [16,17], a well-known autoinflammatory disorder. The LC3-binding adaptor protein p62 (known as SQSTM1/sequestosome-1) binds ubiquitinated substrates, serving as a molecular bridge for their delivery to the autophagosomes, and then promotes their degradation through a proteasomal pathway [3,18].

To test whether the genetic polymorphisms of autophagy signaling confer a predisposition to AOSD, we compared the variations of ATGs between patients with AOSD and healthy participants. We also examined the levels of ATG mRNA expression and serum cytokines and evaluated their correlations with genetic variants in patients with AOSD. In addition, we investigated the association of ATG genotypes with clinical manifestations and disease courses in AOSD patients.

## 2. Materials and Methods

### 2.1. Participants

This prospective case-control study enrolled 129 consecutive patients with AOSD fulfilling the Yamaguchi criteria [19]. Patients with infections, malignancies, or other rheumatic diseases were excluded. The disease activity of each patient with AOSD was assessed using the modified Pouchot score described by Rau et al. [20]. All patients with AOSD received treatment, including corticosteroids, nonsteroidal anti-inflammatory drugs, methotrexate, hydroxychloroquine, sulfasalazine, cyclosporine, and azathioprine. According to the proposed classification of AOSD disease course [21,22], AOSD patients, followed up for at least 1 year, were classified into two patterns: the *systemic* *pattern,* including monocyclic and polycyclic forms, or the *chronic articular* *pattern* (persistent arthritis involving at least one joint and lasting longer than 6 months). We enrolled 129 ethnically and geographically matched healthy participants as controls; none of them reported any autoimmune diseases. This study was approved by the Institutional Review Board of Taichung Veterans General Hospital (approval number: CF11309) and followed the principles of the Declaration of Helsinki; each participant’s written consent was obtained.

### 2.2. Single-Nucleotide Polymorphism Selection and Genotyping Using Direct Sequencing

We used a single-nucleotide polymorphism (SNP) tagging approach to select genetic variants to investigate the genetic variability in six major mammalian autophagic genes: *Beclin-1*, *ATG5*, *ATG7*, *MAP1LC3B (LC3-II*), *SQSTM1*, and *ATG16L1*. To select the most representative SNPs by capturing the majority of genetic variations, SNP genotype information was downloaded from the HapMap database (http://www.hapmap.ncbi.nlm.nih.gov/, accessed on 11 June 2014) and the National Center for Biotechnology Information dbSNP database (http://www.ncbi.nlm.nih.gov/snp, accessed on 11 June 2014). Tag SNPs were selected for *Beclin-1*, *ATG5*, *ATG7*, *SQSTM1*, and *ATG16L1* using the criterion of minor allele frequency ≥10%. We identified 12 tag SNPs from six core autophagy pathway genes based on the relevant literature [23,24,25,26,27,28,29,30] (Supplementary Table S1). Genomic DNA samples were extracted from the peripheral blood of the enrolled patients using Genomic DNA Extraction Kits (RBC Bioscience, New Taipei City, Taiwan). For quality control, we randomly selected 10 samples for duplicates, and the concordance rate was >0.95 for all SNPs assayed. Any SNP that was triallelic (*n* = 1), had little difference in the allele frequency between AOSD and controls (*n* = 2), or demonstrated a Hardy–Weinberg equilibrium *p*-value of <0.01 in the control participants (*n* = 3) was removed. Thus, six SNPs (one of *BECN1*, one of *ATG5*, one of *SQSTM1*, and three of *ATG16L1*) were included in the direct sequencing (Table 1) and further statistical analyses. TaqMan SNP analyzer (Applied Biosystems, Foster City, CA, USA) was used in direct sequencing: rs10512488 of the *BECN1* gene region (TaqMan assay: C_27102741_10), rs573775 of the *ATG5* gene region (C_910347_20), rs565280 of the *SQSTM1* gene region (C_645001_20); rs2241880, rs10210302, and rs1045100 of the *ATG16L1* gene region (C_9095577_20, C_30179764_10, and C_8741775_20, respectively). The polymerase chain reaction (PCR) assay was performed as following steps: 10 min at 95 °C, followed by 40 cycles of 15 s at 95 °C, and 1 min at 60 °C; the PCR buffer used was FastStart Universal Probe Master (ROX) (Roche Life Science, Indianapolis, IN, USA).

### 2.3. Determination of the mRNA Levels of ATGs Using Quantitative PCR

Peripheral blood mononuclear cells (PBMCs) were immediately isolated from the patients’ blood samples by using the Ficoll-Paque PLUS (GE Healthcare Biosciences, Uppsala, Sweden) for density gradient centrifugation. Total RNA was obtained from PBMCs by using the guanidinium isothiocyanate method [31]. Next, 2.5 μg of RNA aliquot was reverse-transcribed using 200 U of Moloney murine leukemia virus reverse transcriptase (Fermentas, Thermo Fisher Scientific, Pittsburgh, PA, USA). By using direct sequencing, we demonstrated a significant difference in the frequencies of *ATG16L1* genotypes between patients with AOSD and healthy controls. In addition, a close link was noted between ATG16L1 and autophagosome formation, evidenced by LC3-II expression. Therefore, the mRNA expression levels of ATG genes, including *MAP1LC3B* (LC3-II) and *ATG16L1*, were determined using Roche FastStart Universal SYBR Green Master Mix (Roche Life Science, Indianapolis, IN, USA). The primer sequences were as follows: for *LC3-II (MAP1LC3B*), 5′-AAGGCGCTTACAGCTCAATG-3′ (forward), and 5′-CTGGGAGGCATAGACCATGT-3′ (reverse); for *ATG16L1*, 5′-ACTGCCTTGGAGGGAAAACT-3′ (forward) and 5′-GCTGCTTCTGCAAGCT CTTT-3′ (reverse); and for *GAPDH*, 5′-GAAGGTGAAGGTCGGAGTC-3′ (forward) and 5′-GAAGATGGTGATGGGATTTC-3′ (reverse). To standardize the mRNA expression levels of the ATGs, the mRNA levels of the housekeeping gene *GAPDH* were also determined in parallel for each sample. The mRNA expression levels of the ATGs were calculated using the comparative threshold cycle (Ct) method and evaluated using 2**^−^^△△^**^Ct^, where △△Ct = patient (Ct_ATGs_ − Ct_GAPDH_) − mean of controls (Ct_ATGs_ − Ct_GAPDH_).

### 2.4. Determination of Serum Levels of Proinflammatory Cytokines Using Enzyme-Linked Immunoabsorbent Assay

Serum IL-1β levels were determined using enzyme-linked immunoabsorbent assay (ELISA; RayBiotech, Norcross, GA, USA), and TNF-α, IL-6, and IL-18 levels were also determined using ELISA (Medical & Biological Laboratories, Nagoya, Japan) according to the manufacturer’s instructions.

### 2.5. Statistical Analysis

Demographic results are presented as the mean ± standard deviation or median (interquartile range [IQR]). For the genotyping of the six candidate genes, we compared the allele or genotype frequencies between AOSD cases and healthy controls; the SNP associations were examined using Fisher’s exact test and rank-ordered according to the lowest *p*-value; *p <* 0.05 was considered statistically significant. The odds ratios (ORs) were calculated as described in a previous study [32].

We performed Kolmogorov–Smirnov test and Shapiro–Wilk test for normality test. The p-value of mRNA expression levels of LC3-II (MAP1LC3B), ATG16L1, and cytokine levels between AOSD patients and health controls were less than 0.05. Therefore, the Mann–Whitney U test was used for between-group comparisons. The association of significant haplotypes or clinical manifestations in patients with AOSD with different disease outcome patterns was examined using multivariant regression.

## 3. Results

### 3.1. Clinical Characteristics of Patients with AOSD

Among 129 patients with AOSD (mean age at study entry, 37.5 ± 14.6 years; 94 women and 35 men) enrolled in the genotyping, the presence of spiking fever (≥39 °C), evanescent rash, sore throat, arthralgia or arthritis, lymphadenopathy, and liver dysfunction were observed in 125 (96.9%), 108 (83.7%), 101 (78.3%), 76 (58.9%), 55 (42.6%), and 47 (36.4%), respectively. However, no significant age or gender differences were observed between patients with AOSD and healthy controls (34.7 ± 10.4 years; 99 women and 30 men). Demographic data were summarized in Supplementary Table S2.

### 3.2. Genotypes, Allele Frequencies, and Haplotypes: Comparing Patients with AOSD and Healthy Controls

**Figure 1:**
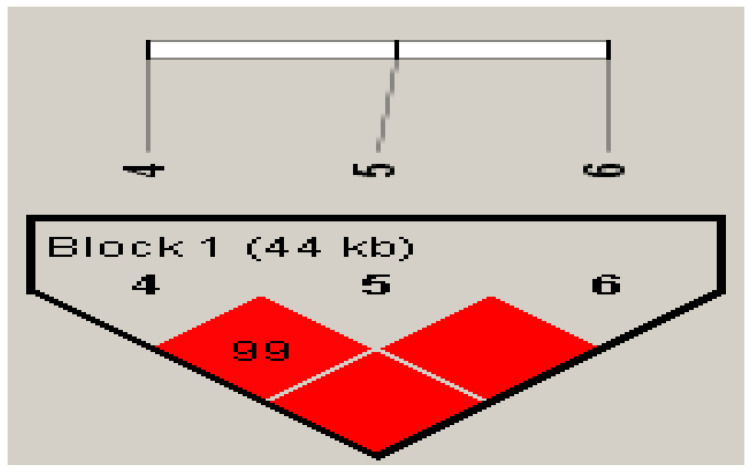
The linkage disequilibrium (LD) plot of the 3 SNPs of the ATG16L1 gene using Haploview with confidence bounds color scheme. All SNPs were in strong LD.

The statistical significance of genotype frequencies when comparing patients with AOSD and controls is summarized in Table 2. All six SNPs in AOSD cases and controls had Hardy–Weinberg equilibrium *p* values of >0.05. A significantly lower frequency of the rs2241880/GG genotype was detected in patients with AOSD than in healthy controls (5.4% vs. 17.1%, *p* = 0.005). However, no significant between-group differences were observed in the distribution of the allelic frequencies of the rs2241880, rs10210302, or rs1045100 variants of *ATG16L1*. These three SNPs were within a block as presented in Figure 1, rs2241880–rs10210302 (D’ = 1, *R^2^* = 1.00), rs2241880–rs1045100 (D’ = 1, *R^2^* = 0.97), and rs10210302–rs1045100 (D’ = 1, *R^2^* = 0.97). Given the significant linkage disequilibrium (LD) of three SNPs of *ATG16L1*, we further examined the association of *ATG16L1* haplotypes with the susceptibility to AOSD (Table 2). Taking the AA/CC/TT haplotype as the reference, we observed an increased OR (95% confidence interval) of the AG/CT/CT haplotype in patients with AOSD compared with the healthy controls (OR 1.74, CI (1.02–2.95), *p* = 0.04).

### 3.3. Association of ATG16L1 Polymorphisms with ATG mRNA Expression Levels and Serum Cytokine Levels in Patients with AOSD

**Figure 2:**
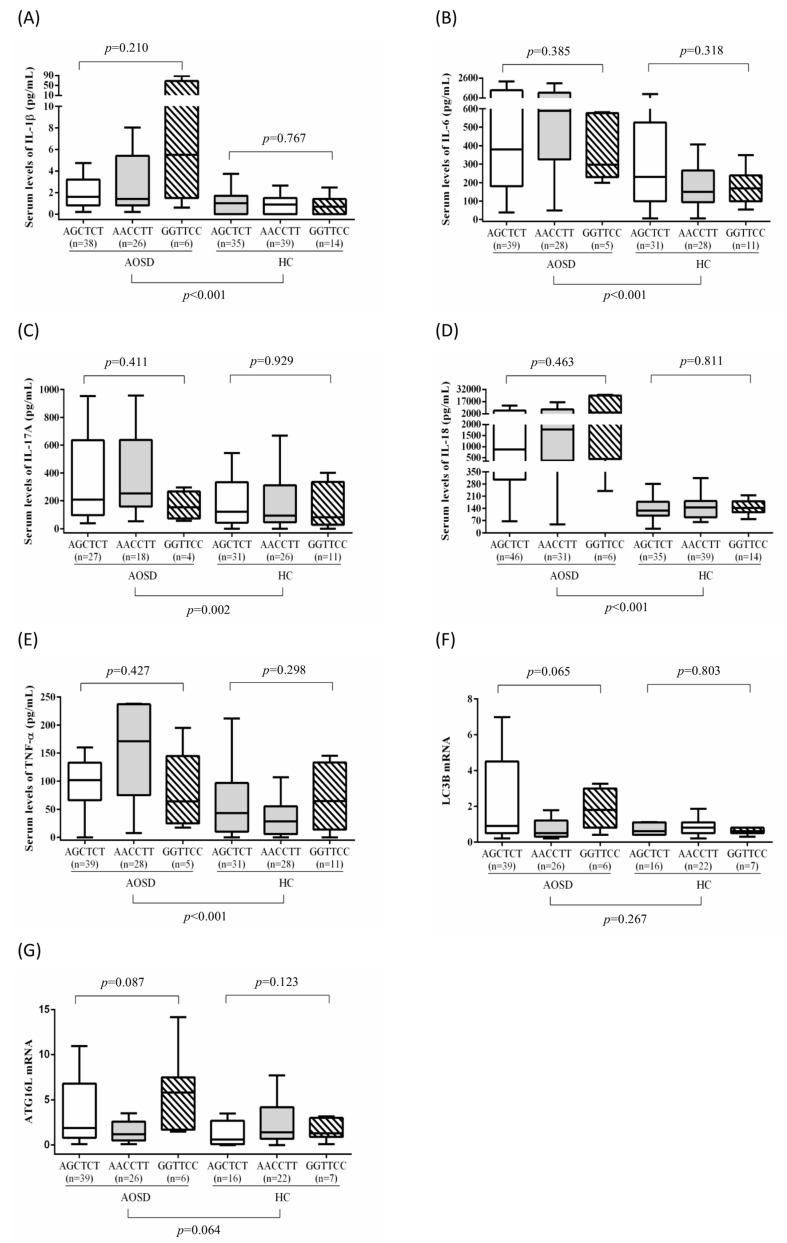
(**A**–**G**) Serum levels of IL-1β, IL-6, IL-17A, IL-18, and TNF-α and mRNA levels of LC3-II and ATGL16L1 in patients with AOSD and healthy controls with different haplotypes.

Because the autophagic process is regulated by ATGs, we examined the functional association of *ATG16L1* haplotypes by determining ATG mRNA expression levels and serum cytokine levels in 83 patients with AOSD and 88 controls from whom examined samples were available. According to a previous study, the G allele of *ATG16L1* rs2241880 was significant in susceptibility to other autoinflammatory diseases, such as Crohn’s disease [33,34]. Therefore, we divided patients with AOSD into two groups by the presence of the G allele of *ATG16L1* rs2241880: GG/TT/CC plus AG/CT/CT and AA/CC/TT based on the haplotypes of rs2241880G/A, rs10210302T/C, and rs1045100C/T. As shown in Table 3, significant differences in LC3-II mRNA expression were noted between the groups in AOSD patients (median 1.1, IQR 1.1–4.3 vs. median 0.5, IQR 0.5–1.2, *p* = 0.026). Though no significant difference was observed, ATG16L1 mRNA expression levels were higher in the GG/TT/CC plus AG/CT/CT group (median 2.0, IQR 2.0–8.1) than in the AA/CC/TT group (median 1.2, IQR 1.2–2.6, *p* = 0.088). As presented in Figure 2, significantly higher serum levels of IL-1β, TNF-α, IL-6, and IL-18 were observed in patients with AOSD (median, 1.65 pg/mL, IQR 0.79–4.09 pg/mL; 183.93 pg/mL, IQR 78.06–573.84 pg/mL; 495.08 pg/mL, IQR 222.43–1138.03 pg/mL; and 1082.82 pg/mL, IQR 347.22–7173.25 pg/mL, respectively) compared with healthy controls (0.87 pg/mL, IQR 0.00–1.45 pg/mL; 43.22 pg/mL, IQR 9.70–88.99 pg/mL; 178.07 pg/mL, IQR 97.83–333.09 pg/mL; and 138.12 pg/mL, IQR 95.28–179.79 pg/mL, respectively; all *p* < 0.001).

Serum TNF-α, IL-6, and IL-18 levels were nonsignificantly higher in patients with AOSD carrying the AA/CC/TT haplotype (median, 219.0 pg/mL, IQR 219–581.3 pg/mL; 589.2 pg/mL, IQR 589.2–1138 pg/mL; and 778.9 pg/mL, IQR 1778.9–7418 pg/mL, respectively) compared with those carrying the GG/TT/CC or AG/CT/CT haplotype (median, 143.3 pg/mL, IQR 143.3–573.8 pg/mL; 354.4 pg/mL, IQR 354.4–1155.1 pg/mL; and 945.0 pg/mL, IQR 945–6632.9 pg/mL, respectively).

### 3.4. Association of Disease Outcomes with ATG16L1 Polymorphisms and Clinical Manifestations in Patients with AOSD

We observed that patients with AOSD carrying the AA/CC/TT haplotype had a significantly higher proportion of skin rashes and a lower proportion of arthritis compared with those with the GG/TT/CC and AG/CT/CT haplotype (95.8% vs. 75.9% and 37.5% vs. 70.9%, *p* < 0.05).

Among patients with AOSD, 98 (76%) exhibited the systemic pattern, and 31 (24%) had the chronic articular pattern of disease outcome. After excluding two patients who did not match the LD, we included 127 patients with AOSD in the analysis of the association of *ATG16L1* haplotypes with disease outcomes. Among 98 patients with the systemic pattern, 5 (5.1%) had the GG/TT/CC haplotype, 51 (52.0%) had the AG/CT/CT haplotype, and 42 (42.9%) had the AA/CC/TT haplotype.

Compared with patients with the chronic articular pattern, patients with AOSD with the systemic pattern had significantly higher ferritin levels (median 1769 μg/mL, IQR 641.5–7525 μg/mL vs. median 972 μg/mL, IQR 267–2425 μg/mL, *p* < 0.05), significantly higher serum IL-18 levels (median 2023.5 pg/mL, IQR 389.7–8020.8 pg/mL vs. 567.6 pg/mL, IQR 242.8–1262.5 pg/mL), and significantly higher ATG16L1 expression levels (median 2.5, IQR 1.20–7.8 vs. median 0.7, IQR 0.2–1.5, *p* = 0.001). However, no significant differences were observed in LC3-II expression levels or serum levels of IL-1β, TNF-α, or IL-6 between the two patient subgroups.

As illustrated in Table 4, multivariate regression analysis indicated that the AA/CC/TT haplotype was a positive predictor of the systemic pattern (OR 3.25, 95% CI 1.15–9.14, *p* = 0.026). Elevated alanine transaminase and C-reactive protein levels were also associated with increased risk of the systemic pattern (OR 1.03, 95% CI 1.00–1.05, *p* = 0.018). However, the occurrence of skin rash, arthritis, sore throat, lymphadenopathy, hepatosplenomegaly, leukocytosis, disease activity scores, and levels of ferritin, IL-1, IL-6, IL-17A, IL-18, TNF-α, LC3-II mRNA, or ATG16L mRNA expression was not predictive of the systemic pattern. We also performed logistic regression analysis for the chronic articular pattern. Neither clinical manifestations nor biomarkers above showed significance in the prediction of a chronic articular pattern.

**Figure 3:**
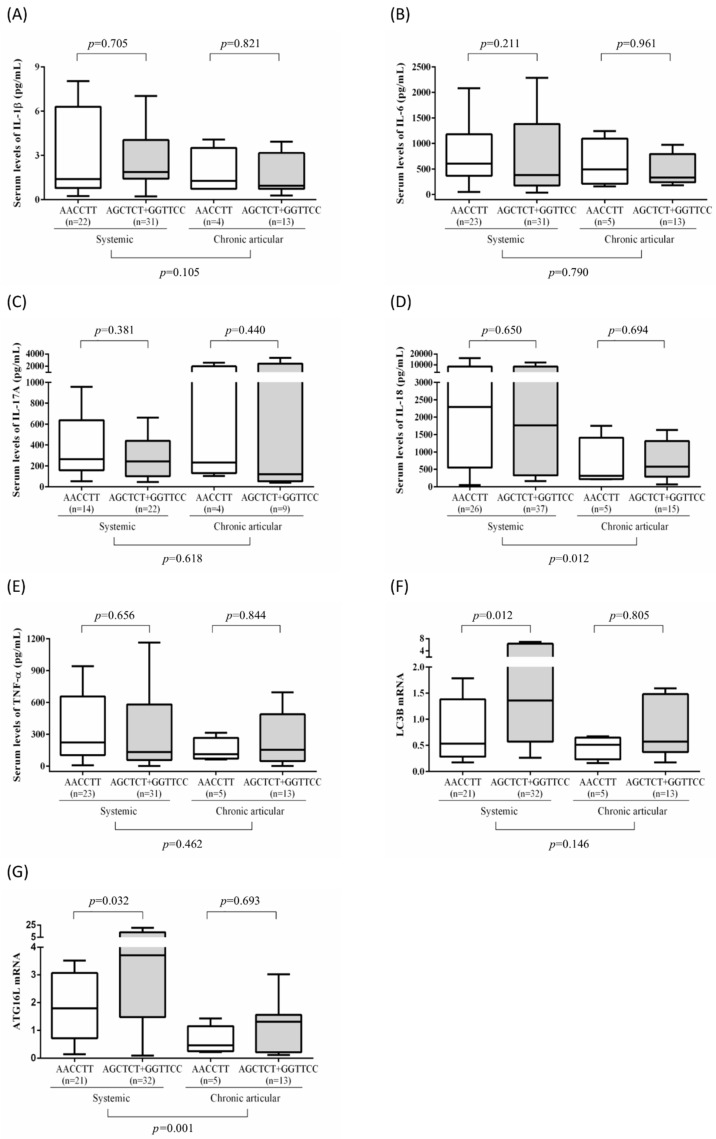
(**A**–**G**) Serum levels of IL-1β, TNF-α, IL-6, IL-17A, and IL-18, and mRNA levels of LC3-II and ATGL16L1 in patients with AOSD carrying different haplotypes.

We further analyzed the correlation between mRNA expression levels or serum cytokine levels and *ATG16L1* haplotypes in patients with ASOD. As illustrated in Figure 3, significantly higher levels of ATG16L1 and LC3-II expression were observed in the AGCTCT plus GGTTCC haplotypes (median 3.7, IQR 3.7–13.4 and 1.4, IQR 1.4–6.4, respectively) compared with that in the AACCTT haplotype (median 1.8, IQR 1.8–3.1 and 0.5, IQR 0.5–1.4, respectively) in the systemic pattern subgroup. However, no significant differences in serum cytokine levels were observed between the haplotype subgroups in patients with the systemic pattern of AOSD. Moreover, no significant differences in serum cytokine levels, ATG16L1 expression levels, or LC3-II expression levels were observed between haplotypes in patients with the chronic articular pattern of AOSD.

## 4. Discussion

The present study is the first to investigate the associations of ATG polymorphisms with disease susceptibility, clinical manifestations, and disease outcomes in patients with AOSD, which is a rare autoinflammatory disease [35,36]. We identified a valuable genetic variant, the AA/CC/TT haplotype of the *ATG16L1* gene composed of three SNPs (rs2241880, rs10210302, and rs1045100), as a genetic factor that is significantly associated with the systemic pattern of AOSD by using direct sequencing. Regarding its functional association, lower mRNA expression levels of LC3-II and ATG16L1 were noted in patients with AOSD carrying the AA/CC/TT haplotype than in those with the AGCTCT plus GGTTCC haplotype. Given that ATG16L1 deficiency induces IL-1β and IL-18 production [37], patients with AOSD carrying the AA/CC/TT haplotype may have impaired autophagy and consequently high-grade systemic inflammation, as demonstrated by our recent study [8]. However, our results should be validated in large cohorts of patients with AOSD of different ethnicities. The *ATG16L1* gene on 2q37.1 encodes a short coiled-coil protein that interacts with ATG5 and ATG12 to form a 350-kDa multimeric complex that plays a crucial role in autophagosome formation [15]. Consistent with this finding, our previous study revealed increased levels of the autophagosome, ATG, and LC3-II expression in patients with AOSD compared with controls [8]. In the present study, the frequencies of the GG/TT/CC or AG/CT/CT haplotype of *ATG16L1* in patients with AOSD were significantly different from those of controls, suggesting that *ATG16L1* gene polymorphisms are associated with AOSD susceptibility. Among patients with AOSD, those carrying the AA/CC/TT haplotype had the lowest level of ATG16L1 and LC3-II mRNA expression. Our results support the findings of a study demonstrating that ATG16L1-deficient macrophages increased IL-1β and IL-18 levels [37]; additionally, Choe et al. revealed that macrophages transfected with ATG16L1 siRNA under the stimulation of monosodium urate crystals exhibited enhanced IL-1β expression [38]. These observations suggest that patients with AOSD carrying the AA/CC/TT haplotype may have impaired autophagy, leading to high-grade systemic inflammation.

AOSD has diverse manifestations. Although Fc RIIa and Fc IIIa polymorphisms have been associated with elevated ferritin levels [39] and IL-18 polymorphism has been associated with higher disease activity scores [40], no study had evaluated the association of autophagy gene polymorphisms with clinical manifestations in patients with AOSD. In the present study, patients with AOSD carrying the AA/CC/TT haplotype had a significantly higher proportion of skin rash and a lower proportion of arthritis than those carrying the other haplotypes. However, our results should be validated in a large cohort of patients with AOSD.

The disease courses and prognosis of patients with AOSD vary considerably [22,23], and none of the clinical or laboratory variables can predict disease course. Consistent with a report indicating a difference in serum cytokines between patients with systemic and chronic articular patterns [7], in our cohort, patients with the systemic pattern had significantly higher serum ferritin and IL-18 levels than those with the chronic articular pattern. Consistent with our findings that patients carrying the AA/CC/TT haplotype had high-grade systemic inflammation, multivariate analysis indicated that the AA/CC/TT haplotype was a positive predictor of the systemic pattern. Accordingly, we speculate that *ATG16L1* haplotypes may help predict the AOSD disease course.

This study has some limitations. The sample size may be too small to definitively conclude the associations of *ATG16L1* haplotypes with disease course or clinical outcome. However, a larger sample size may not be easily available because AOSD is a rare disease, with an estimated prevalence of <1 per 100,000 people [35,36], placing it among orphan diseases. Further, only six candidate genetic variants of six core autophagy pathway genes were genotyped in this cohort. In addition, we did not enroll other ethnic groups or evaluate the impact of environmental factors that may influence the manifestation of the genetic phenotypes into disease phenotypes. Future studies should enroll larger cohorts of patients with AOSD of different ethnicities and conduct fine-mapping and functional studies of other identified genetic variants.

## 5. Conclusions

The AA/CC/TT haplotype of the *ATG16L1* gene may be linked to clinical manifestations and the systemic pattern of the disease in patients with AOSD. Patients with the AA/CC/TT haplotype had lower mRNA expression levels of LC3-II and ATG16L1, suggesting a relationship between *ATG16L1* gene polymorphisms and the autophagy pathway in AOSD.

## Figures and Tables

**Table 1:** Single-nucleotide polymorphisms of six-candidate genes potentially involved in the autophagy signaling pathway.

Gene	SNP ID	TaqMan no. Chromosome: Position	Location	MAF	HWE (*p*-Value)
*Beclin-1*	rs10512488	C_27102741_10 17:42811886	intron variant	A (G > A), 0.01	0.751
*ATG5*	rs573775	C_910347_20 6:106316991	intron variant	A (G > A), 0.33	0.586
*SQSTM1*	rs565280	C_645001_20 5:179826926	intron variant	A (G > A), 0.02	0.917
*ATG16L1*	rs2241880	C_9095577_20 2:233274722	transcript variant	G (A > G), 0.34	0.064
	rs10210302	C_30179764_10 2:233250193	transcript variant	T (C > T), 0.34	0.141
	rs1045100	C_8741775_20 2:233294951	transcript variant	C (T > C), 0.33	0.201

ATG: autophagy-related gene; *ATG16L1*: autophagy-related 16-like 1; MAF: minor allele frequency; HWE: Hardy–Weinberg equilibrium.

**Table 2:** Direct sequencing demonstrated that the frequencies of *ATG16L1* polymorphisms and haplotypes were associated with susceptibility to AOSD.

	AOSD (*n* = 129)	HC (*n* = 129)	*p* value	OR (95% CI)		OR (95% CI) ^a^	
ATG16Lrs2241880			0.001 **				
GG	7 (5.4%)	22 (17.1%)		Reference		Reference	
AG	74 (57.4%)	50 (38.8%)		4.65	(1.85–11.71)	4.72	(1.87–11.90)
AA	48 (37.2%)	57 (44.2%)		2.65	(1.04–6.73)	2.61	(1.02–6.64)
ATG16Lrs10210302			0.001 **				
TT	6 (4.7%)	21 (16.3%)		Reference		Reference	
CT	75 (58.1%)	52 (40.3%)		5.05	(1.91–13.37)	5.14	(1.94–13.65)
CC	48 (37.2%)	56 (43.4%)		3.00	(1.12–8.04)	2.98	(1.11–7.99)
ATG16Lrs1045100			0.003 **				
TT	49 (38.0%)	58 (45.0%)		Reference		Reference	
CT	74 (57.4%)	52 (40.3%)		1.68	(1.00–2.83)	1.74	(1.03–2.93)
CC	6 (4.7%)	19 (14.7%)		0.37	(0.14–1.01)	0.37	(0.14–1.01)
	AOSD (*n* = 127)	HC (*n* = 124)	*p*-value	Logistic regression			
	*n* (%)	*n* (%)		OR (95% CI)		*p* value	
ATG16L1 haplotypes			0.002				
GG/TT/CC haplotype	6 (4.7%)	19 (15.3%)		0.37 [0.14–0.997]		0.049 *	
AG/CT/CT haplotype	73 (57.5%)	49 (39.5%)		1.74 [1.02–2.95]		0.040 *	
AA/CC/TT haplotype	48 (37.8%)	56 (45.2%)		Reference			

AOSD: adult-onset Still’s disease; HC: healthy controls; OR: odds ratio; CI: confidence interval; ^a^ Adjusted for sex. * *p* < 0.05, ** *p* < 0.01.

**Table 3:** A comparison of serum levels of LC3B mRNA, ATG16L mRNA, and inflammatory cytokines between different haplotypes in AOSD patients.

	AOSD						
	AGCTCT + GGTTCC			AACCTT			*p*-Value
	*n*	Median	IQR	*n*	Median	IQR	
LC3B mRNA	45	1.1	(1.1–4.3)	26	0.5	(0.5–1.2)	0.026 *
ATG16L mRNA	45	2.0	(2.0–8.1)	26	1.2	(1.2–2.6)	0.088
Serum levels of IL-1 (pg/mL)	44	1.7	(1.7–4)	26	1.4	(1.4–5.4)	0.947
Serum levels of IL-6 (pg/mL)	44	354.4	(354.4–1155.1)	28	589.2	(589.2–1138)	0.208
Serum levels of IL-17A (pg/mL)	31	180.1	(180.1–559.5)	18	252.5	(252.5–637)	0.272
Serum levels of IL-18 (pg/mL)	52	945.0	(945–6632.9)	31	1778.9	(1778.9–7418)	0.519
Serum levels of TNF-α (pg/mL)	44	143.3	(143.3–573.8)	28	219.0	(219–581.3)	0.529

AOSD: adult-onset Still’s disease; IQR: interquartile range; * *p* < 0.05.

**Table 4:** Logistic regression analysis for the systemic pattern of adult-onset Still’s disease.

	Univariate Analysis			Multivariate Analysis		
	OR	(95% CI)	*p* Value	OR	(95% CI)	*p* Value
Age	0.99	(0.97–1.02)	0.603			
Gender						
Male						
Female	1.00	(0.40–2.53)	0.997			
*ATG16L1*						
AGCTCT + GGTTCC	ref.			ref.		
AACCTT	2.87	(1.08–7.69)	0.035 *	3.25	(1.15–9.14)	0.026 *
Rash	1.07	(0.35–3.22)	0.907			
Arthritis	0.00	(0.00–	0.997			
Sore throat	1.87	(0.74–4.75)	0.188			
Lymphadenopathy	2.32	(0.94–5.74)	0.068			
hepatospenomegaly	2.25	(0.48–10.53)	0.303			
Leukocytosis	1.24	(0.46–3.31)	0.667			
Liver dysfunction	2.76	(1.03–7.38)	0.043 *			
Disease activity score	1.08	(0.76–1.54)	0.662			
ALT	1.03	(1.01–1.05)	0.013 *	1.03	(1.00–1.05)	0.018 *
WBC	1.00	(1.00–1.00)	0.718			
Hemoglobin	0.90	(0.70–1.16)	0.427			
Platelet	1.00	(1.00–1.00)	0.342			
ESR	1.00	(0.99–1.01)	0.747			
CRP	1.07	(1.00–1.15)	0.046 *	1.06	(0.98–1.15)	0.119
Ferritin	1.00	(1.00–1.00)	0.070			
Serum levels of IL-1 (pg/mL)	1.00	(0.97–1.04)	0.957			
Serum levels of IL-6 (pg/mL)	1.00	(1.00–1.00)	0.876			
Serum levels of IL-17A (pg/mL)	1.00	(1.00–1.00)	0.050			
Serum levels of IL-18 (pg/mL)	1.0003	(1.00–1.001)	0.040 *			
Serum levels of TNF-α (pg/mL)	1.00	(1.00–1.00)	0.307			
LC3-II mRNA	1.01	(0.98–1.05)	0.402			
ATG16L1 mRNA	1.00	(1.00–1.00)	0.760			

ATG16L1: Autophagy-related 16-like 1; ALT: alanine transaminase; WBC: White blood cell; ESR: Erythrocyte Sedimentation Rate: CRP: C-reactive protein. Logistic regression; * *p* < 0.05.

## Data Availability

The datasets used and/or analyzed during the current study are available from the corresponding author on reasonable request.

## Supplementary Materials

The following are available online at https://www.mdpi.com/article/10.3390/genes12060904/s1, Table S1: The examined single nucleotide polymorphisms (SNPs) of 12 candidate genes potentially involved in autophagy signaling pathway; Table S2: Demographics and clinical manifestations of AOSD patients and health controls
