# Supplementary material for: The Association of ATG16L1 Variations with Clinical Phenotypes of Adult-Onset Still’s Disease
Source: Genes (Basel). 2021 Jun 11;12(6):904. doi: 10.3390/genes12060904 (PMC8230810; doi:10.3390/genes12060904)
Supplement: Supplementary file 1 [file genes-12-00904-s001.zip › genes-1231078-supplementary.pdf]

Table S1. The examined single nucleotide polymorphisms (SNPs) of 12 candidate genes potentially involved in autophagy signaling pathway

Gene	SNP ID	TaqMan no.	Chromosome: position	Location	MAF	HWE (p-value)
Beclin-1	rs10512488	C_27102741_10	17:42811886	intron variant	A (G>A), 0.01	0.751
ATG5	rs573775	C_910347_20	6:106316991	intron variant	A (G>A), 0.33	0.586
	rs510432	C_910351_10	6: 106326155	intron variant	T (C>T), 0.29	0.239
	rs633724	C_1328947_20	6: 106286165	intron variant	T (C>T), 0.30	0.249
	rs9373839	C_3001898_10	6: 106207742	intron variant	C (T>C), 0.02	0.787
ATG7	rs1375206	C_1288835_10	3: 11297643	intron variant	C (G>C), 0.34	0.000
MAP1LC3B	rs2873702	No available probe	16:87403154	intron variant	A (G>A), 0.00	0.000
SQSTM1	rs565280	C_645001_20	5:179826926	intron variant	A (G>A), 0.02	0.917

	rs4935	C_11416203_10	5: 179833153	synonymous codon	C (T>C), 0.33	0.000
ATG16L1	rs2241880	C_9095577_20	2:233274722	transcript variant	G (A>G), 0.34	0.064
	rs10210302	C_30179764_10	2:233250193	transcript variant	T (C>T), 0.34	0.141
	rs1045100	C_8741775_20	2:233294951	transcript variant	C (T>C), 0.33	0.201

Table S2. Demographics and clinical manifestations of AOSD patients and health controls

	AOSD (n=129)	HC (n=129)
Age, year (Mean± SD)	37.5 ± 14.6	34.7 ± 10.4
Female (n,%)	94 (72.9%)	99 (76.7%)
Fever (n,%)	125 (96.9%)	NA
Rash (n,%)	108 (83.7%)	NA
Sore throat (n,%)	101 (78.3%)	NA
Arthrtis (n,%)	76 (58.9%)	NA
Lymphadenopathy (n,%)	55 (42.6%)	NA
Liver dysdunction (n,%)	47 (36.4%)	NA
